# A survey of the use of electronic scientific information resources among medical and dental students

**DOI:** 10.1186/1472-6920-6-28

**Published:** 2006-05-09

**Authors:** Kalle Romanov, Matti Aarnio

**Affiliations:** 1 Research & Development Unit for Medical Education, University of Helsinki, P.O. Box 63, FIN 00014, Helsinki, Finland

## Abstract

**Background:**

To evaluate medical and dental students' utilization of electronic information resources.

**Methods:**

A web survey sent to 837 students (49.9% responded).

**Results:**

Twenty-four per cent of medical students and ninteen per cent of dental students searched MEDLINE 2+ times/month for study purposes, and thiry-two per cent and twenty-four per cent respectively for research. Full-text articles were used 2+ times/month by thirty-three per cent of medical and ten per cent of dental students. Twelve per cent of respondents never utilized either MEDLINE or full-text articles. In multivariate models, the information-searching skills among students were significantly associated with use of MEDLINE and full-text articles.

**Conclusion:**

Use of electronic resources differs among students. Forty percent were non-users of full-text articles. Information-searching skills are correlated with the use of electronic resources, but the level of basic PC skills plays not a major role in using these resources. The student data shows that adequate training in information-searching skills will increase the use of electronic information resources.

## Background

Over the last several decades studies have shown that the use of computerized information systems by medical professionals can improve the quality of care, enhance the use of evidence-based treatments, and maintain and update knowledge [1,2]. It has also been shown that even though doctors generate various questions during patient care [3], many of these questions remain unanswered because physicians typically search answers to only one third of the questions [4]. MEDLINE, especially, is often considered too demanding to be searched effectively in clinical settings, but there may be solutions available for improving its usability. In recent studies of doctors' use of online evidence, it has been reported that over 80% of practitioners studied believed that the use of electronic information resources has the potential to improve patient care [5,6].

One of the major goals of medical education is to encourage students to maintain their knowledge of medical science by becoming life-long learners. Adequate skills in information seeking and regular use of original scientific sources are key elements in this process. With regard to medical informatics education, both information processing and information technology have been considered relevant for the quality of healthcare [3]. Additionally, it is believed that medical students need training to learn how to use Web-based search tools and techniques in order to find high-quality information resources [7].

Some current studies explore how medical and dental students at various levels of the curriculum utilize electronic scientific resources for medical information. Virtanen reported in 2000 that younger dental students at the University of Oulu in Finland in general were more accustomed to utilizing information resources for educational purposes than senior dental students [8]. Compared to working clinicians, students are probably more prone to use computer-based information resources in their studies and clinical interactions. Recently, Peterson et al. reported that the majority of medical students preferred electronic sources as primary resources, and exclusively electronic textbooks with rapid searching capabilities were the most frequently utilized [9]. Availability of full-text articles and other databases may have a major impact on the selection of information resources among students. In one study of the utilization of library resources among medical students, full-text articles online were the single most valued resource [10].

The aim of our study was to find out how electronic information resources are utilized by medical and dental students and what specific trends may be found among students at different years of study. We were also interested in assessing the relationship between the use of electronic information resources and computer literacy.

## Methods

The Medical Faculty of Helsinki University offers a six-year medical curriculum made up of three phases: a two-year preclinical phase, a one-term clinical-theoretical phase, and a three-year clinical phase. The dental program consists of two years of preclinical theory taken with medical students, and a three-year clinical phase. Students have an obligatory 10-credit part of the study program, which is tailored to give an introduction to scientific work. The module ends with a written scientific essay based on research that the students have conducted or a scientific review of a given topic.

The central medical library, 'Terkko', of Helsinki University [11] is located on the campus and currently provides about 10,000 online journals and about 3,800 printed journals. About half of the online journals are from the field of medicine. The major component of the electronic library information system of Terkko is Vertex Online, an integrated information service that covers medicine and health sciences. Vertex combines bibliographic databases with electronic full-text journals. The service provides access to such databases as MEDLINE integrated with Journals@Ovid Full Text-journals. Also available are the Evidence-Based Medicine databases, OVID-MEDLINE, Journal Navigator for all full-text journals, Cochrane library, and electronic medical textbooks published online (including Harrison's Online and about fifty other medical eBooks of various specialities). In addition, students have access to the Finnish health portal Terveysportti [12], a national medical internet-service with an extensive collection (over 1,200 titles) of concise EBM guidelines for primary care, evidence summaries, other medical and dental databases, a database of the most important Finnish medical journals, a database of pharmaceutical information, and several databases of classification and coding systems in health care. Terveysportti is equipped with rapid advanced searching capability and extensive use of hyperlinks among related topics.

On campus, the medical faculty provides over 100 student work stations, of which almost half can be accessed around-the-clock for electronic library resources. The location of the activities of the Institute of Dentistry is two kilometers away from the medical campus where the central medical library is located. Approximately 30 student work stations are available for dental students to use electronic resources. There is a separate library of dentistry in the Institute of Dentistry.

As part of the curriculum, during the first term both medical and dental students participate in a course in computer facilities and informatics, which presents principles of searching scientific literature to obtain information from MEDLINE and the Internet for study and for research (1.2 credits). The course contains practical training and tasks in searching MEDLINE and the Web for medical information on various topics. During the spring term of the third year, medical students are taught the basics of medical informatics, including advanced use of MEDLINE and other clinical databases for gathering relevant information for clinical purposes (0.5 credits). The dental students do not have a similar informatics course in their curriculum.

The student data were collected in November 2004 using an electronic questionnaire to assess the use of scientific information sources among medical and dental students at Helsinki University (Table 7). By means of e-mail lists, the survey was addressed to 837 medical and dental students, of which 418 (49.9%) responded during the two-week survey period (Table 1). Two reminder messages were used to promote response.

An e-mail containing a link to the questionnaire URL was sent to the students, who were queried about such background information as course status, gender, use of computers (hrs/week), and availability of Internet connections at home. Students were also asked to assess separately their weekly hours of computer use on campus vs. at home. To determine the online use of information resources in electronic form, a survey was developed. A pilot test of the survey was done with a group of volunteers, and the wording of some items was changed as a result. The frequency of using different information resources in electronic form was self-assessed on a four-point scale ranging from 1 (for "not at all") to 4 (for "at least twice weekly"). Among the choices on the questionnaire were searching (I) MEDLINE database separately for study and research, (II) the Finnish medical portal 'Terveysportti', (III) the Cochrane library, (IV) electronic full-text articles, (V) electronic textbooks, and (VI) medical information from the World Wide Web. Additionally, students were asked to rate their various skills in information searching and in using PCs (a range of computer applications was based on skills recommended by the medical faculty). A four-point scale was used ranging from 0 (for 'don't know how to use it') to 3 (for 'my skills are OK'). When respondents did not rate their performance level, but indicated instead that they never used an application, their response were recoreded as '0'. To perform data reduction the skills were analyzed with a factor analysis (principle component analysis; oblimin rotation with Kaiser normalization), which produced two, well-defined factors containing nine items of '*Basic PC skills' *(word processing, e-mail, use of the Web etc; coefficients in the final solution were in the range 0.707–0.795, Crohnbach alpha .87) and seven items of '*Search skills' *(MEDLINE, Cochrane library, Terveysportti medical portal, and using full-text skills; coefficients in the final solution were in the range 0.608–0.752, Crohnbach alpha .79). The scores of items were tallied to form the two scales mentioned above. The mean score of the scale 'Basic PC skills' was 24.4 (SD 9.6, min 2, max 45) and that of the 'Search skills' 17.9 (SD 6.9, min 0, max 33), respectively. These two scales measured skills that were moderately correlated with another (r = .61). Included in the questionnaire was a list of additional computing applications of information seeking/processing. Students were asked to report on which topic they would be sufficiently interested in to participate in a training program for improving their skills in information seeking/processing and using various electronic resources in studies (e.g., the use of full-text articles, electronic textbooks, searching MEDLINE, PDF-file management, and how to maximize usability of electronic information resources at home). The responses (0 for 'no', 1 for 'yes') were totaled to give a scale of readiness for further training (mean 3.2, SD 2.3, min 0, max 12).

Statistical differences of 'Basic PC Skills' and 'Search Skills' means were tested with t-test or Mann-Whitney U-test. We studied the association of medical students' use of electronic information resources (use of MEDLINE in studies or research, and use of full-text articles) with 'Basic PC Skills' and 'Search Skills'. The use of electronic information resources was treated as a binary outcome variable (0 = no, 1 = yes). 'Basic PC Skills' and 'Search Skills' were primary explanatory variables in the binary logistic regression model (SPSS 12.1 statistical software). To evaluate independent effects of primary explanatory variables all models were adjusted for gender, duration of study in years, and student status (medical/dental). 'Basic PC Skills' and 'Search Skills' were divided in tertiles separately for medical and dental students (lowest/intermediate/highest level). In the statistical analyses, we compared the intermediate and highest levels of skills to the lowest. The first-year students were excluded from the models, since their computing course required use of MEDLINE and full-text articles. The logistic model allows primary explanatory variables to be intercorrelated and simultaneously estimates the association with the outcome variable.

## Results

Among 418 respondents, eighty-three per cent of medical and eighty-four per cent of dental students had a computer with Internet connections at home. Thirty-four per cent of students used a home computer for studying four or more hours per week. Twenty-five per cent of students used campus computers for studying four or more hours per week. There was a significant correlation between the working hours with a PC at home and working hours with a campus computer (r = 0.198, p < 0.001).

After the second year of study, the medical students scored higher on scores of 'PC Skills' and/or 'Search Skills' as compared to dental students. These differences were significant between medical and dental students among the third-, fourth- and fifth-year students (Table 2).

Twenty-four per cent of the medical and twenty per cent of the dental students searched information from MEDLINE twice a month or more frequently for study purposes (Table 3). The proportion of regular users for study purposes was highest during the first year and the last two years of study both among medical and among dental students. Thirty-two per cent of the medical and twenty-four per cent of the dental students searched information from MEDLINE for their own scientific work twice a month or more. There was a rising trend of use for scientific work through the study years in both groups. Thirty-three per cent of medical and ten per cent of dental students read full-text articles at least 2–7 times/month (Table 3). Thirty-nine per cent of the medical and forty-eight per cent of the dental students never used electronic full-text articles.

The score of search skills was moderately correlated with the score of PC skills (r = .606) (Table 4). The correlations between the score of search skills and the use of MEDLINE for study (r = .379) or research (r = .379) were very similar. The correlation between the score of PC skills and the use of MEDLINE for study or research was lower (r = .171 – .184). The correlation coefficient between the use of full-text articles and the score of search skills was higher (r = .459) compared to the coefficient with the score of PC skills (r = .204) (Table 4).

The logistic model analysis was performed to simultaneously estimate the association of the score of search skills and the score of PC skills with the three outcome variables. The score of search skills was significantly associated with the use of MEDLINE for study or research (Table 5). In this relationship there was a dose/response effect; the higher the score in search skills, the higher the probability MEDLINE was used frequently. Similar results were found in the use of full-text articles (Table 5). Furthermore, basic PC skills were also significantly associated with the use of MEDLINE for research. No significant effects of gender were found, nor were there significant differences between medical and dental students.

Altogether, there were 49 students who did not utilize either MEDLINE or full-text articles. Thirty-two (eleven per cent) medical students and twelve (fifteen per cent) dental students did not use either MEDLINE or full-text articles at all. The forty-nine non-users had significantly lower scores on the totaled scale 'PC Skills' compared to students who utilized these resources (mean 19.6; SD 9.3 vs. 24.9; SD 10.8, p < 0.001, t-test); correspondingly, the same results were obtained on the totaled scale 'Search Skills' (mean 11.9; SD 7.0 vs. 18.6; SD 6.5, p < 0.001, t-test). The non-users also expressed significantly less interest in further training in information retrieval, measured with the totaled scale of applications to be further studied, gaining a median of 2.0 vs. 3.0 (Mann Whitney U-test, p = 0.001). The non-users also used other information resources less compared to other students. Their use of Cochrane library was at least twice/month 0 per cent vs. 7.0 percent among other students, respectively, the use of electronic textbooks at least twice/month 0 per cent vs. 12.1 percent, and the use of web at least twice/month 30.6 per cent vs. 45.9 per cent.

Results of using other electronic databases (e.g., the Cochrane library), the medical portal Terveysportti, electronic textbooks of the medical library, and searching the Web for other resources of medical information are shown in Table 6. On average, thirty per cent of the medical students and seven per cent of the dental students searched information frequently from the major medical portal 'Terveysportti'. Among medical students, the use of Terveysportti and Cochrane library steadily increased to the end of their studies. The use of other medical databases was quite the same in all courses, as was also the utilization of the Web. In the utilization of electronic textbooks, there were two to three-fold differences between courses.

## Discussion

MEDLINE is widely considered the primary source for biomedical journal literature. It is evident that MEDLINE as a bibliographic database containing references to journal articles can provide only a fraction of the information available in the original article. Consequently, searching MEDLINE does not mean that the articles are also read. Because article abstracts are not always a sufficient source of information, the extent of utilization information in the form of full-text documents is an interesting issue when considering the dedication of students to learning the core issues of the curriculum.

In our material, medical students used MEDLINE more often than dental students, both for study and research. The high percentage among the first-year students was a likely result of the course in informatics, which required the use of MEDLINE. Frequent users of full-text articles were clearly more prevalent among medical students than among dental students. Our data does not provide information about what proportion of full-text articles is used for a student's research activities and what proportion is for study. Our data allowed us only to estimate what proportion of students are non-users of full-text articles. Thirty-nine per cent of medical students and forty-eight per cent of dental students did not use full-text articles at all. This seems to be an interesting finding and should be taken into account for developing both medical and dental curricula. We recommend that students should have more practical training in information seeking as well as in reviewing and interpreting the results of scientific papers. This training should be integrated into the current clinical topics in each year of study, perhaps with the PBL (Problem Based Learning) -method, to encourage students to use all the information in scientific papers that is available in full-text, not just the abstracts. We found that the major predictor for using MEDLINE and full-text articles is information-searching skills. Furthermore, in the multivariate model, general computer literacy seemed to be a significant factor in the use of MEDLINE for research. This finding could be interpreted with a positive correlation between the research activities of a student and his/her basic PC skills. Presumably, those students who are intensively involved with research do extensive MEDLINE searching, and simultaneously they also possess several other computing skills. Basic PC skills did not seem to be associated significantly with students' use of full-text articles.

The observed differences in the use of resources between medical and dental students are at least partly due to differences in the curriculum after the second year of study. Medical students scored higher on the scale 'Search Skills' compared to dental students. This finding can be at least partly explained by the fact that in the medical curriculum there is a separate informatics course in the third year, which seems to supply the skills for searching information effectively. The lower score of dental students on the scale of 'PC Skills' is most evident among third- to fifth-year students, indicating the additional training needs of senior dental students.

We also collected data in the use of other information resources, which are probably more clinically oriented and more usable for practical clinical information. Peterson et al. reported that UpToDate, an electronic clinical reference, was the most frequently used information resource among medical students in Iowa [9]. The authors also speculated that medical students use electronic information resources much more than has been reported among practicing physicians, but they did not demonstrate this speculation with empirical data. In a recent study in Nevada, the abilities of practicing physicians and medical students to search Boolean queries were compared, and both demonstrated deficiencies in optimal phrasing. The students were more actively using MEDLINE than the physicians [13]. The frequency of 'Terveysportti' use seemed to climb rapidly from the third year to the sixth. Thus it is possible that during the study period, students gradually adapt to using the electronic resources more and more frequently and finally, during the last year of study develop to a level that seems to outpace younger students. The Finnish health portal 'Terveysportti' is frequently used by primary health doctors nationwide, too [14].

The proportion of other database users (e.g., the Cochrane library) among students was quite high, but the dichotomization of students into non-users vs. others may give slightly too positive an impression. The distribution of answers was quite skewed, and the proportion of regular users was very low (six per cent). In previous studies, medical students have not rated Web sites as being particularly useful for medical information [9,10], and our data gave quite similar results: only a small minority of fifth- and sixth-year students regularly searched the Web for information. This result can be at least partly explained by the difficulty in finding high quality resources on the Internet.

When interpreting our results, some confounding factors need to be considered. First, we collected our data by means of an electronic survey, which may have biased our results. Previously, it has been reported that electronic survey response may be affected by the level of use of electronic communication technology overall [15]. Furthermore, the response rate of the survey was only modest, which may accentuate this bias. Our result may overestimate the usage of electronic resources by students, since the low-users of electronic resources may be underrepresented in our material, which was collected by means of electronic survey [15]. We can assume that the responders to this study represent those individuals who utilize electronic information resources with similar levels of activity. Additionally, some other limitations need to be considered in interpreting the results of this study. First, the material of our study among medical and dental students is limited to one medical faculty in which the utilization of electronic resources may have characteristics of its own. We did not obtain objective data on the use of resources, and we did not inquire about usage of traditional, printed forms of scientific articles, which may be frequently read by some subgroups of students. Also the extent of use of course textbooks and other printed learning materials may have contributed to the outcome. It is also a limitation that we did not ask students to grade the overall usefulness of various resources or to show their preferences for various information resources.

## Conclusion

On the whole, only one-third of medical students and one-tenth of dental students were regular users of full-text articles. However, the use of full-text articles increases moderately toward the end of study among both medical and dental students, which is a trend similar to the use of MEDLINE for research. These findings may be explained by the fact that the students are doing more independent work (e.g., theses) at the end of their studies. During the latter half of the study period students also need more intensive clinical perspective on various topics, which is attained through clinical training and careful reading of articles about clinical research. Another explanation for the outcome is that the students become more familiar with the use of biomedical information sources during their years of study.

In order to support the use of primary scientific information resources, the use of full-text articles should be encouraged in the medical curriculum. Additionally, student skills in searching references from databases, and reading full-text articles should be improved with a revised training program. The level of basic PC skills does not seem to be an important factor in students' use of electronic scientific resources.

## Competing interests

The author(s) declare that they have no competing interests.

## Authors' contributions

MA participated in the design of the study and carried out the data collection. KR participated in the design of the study, carried out the data and statistical analysis, and drafted the manuscript. Both authors read and approved the final manuscript.

## Pre-publication history

The pre-publication history for this paper can be accessed here:



## Figures and Tables

**Table 1:** The number of medical (M)/dental (D) students and response rate by year of study

**Year of study**	**M1**	**D1**	**M2**	**D2**	**M3**	**D3**	**M4**	**D4**	**M5**	**D5**	**M6**	**All D**	**All M**	**Total**
N	67/106	24/40	60/123	17/35	55/121	14/32	44/112	19/37	32/115	7/23	37/93	81	295	418^1^
Response rate	63.2%	60.0%	48.8%	48.6%	45.4%	43.8%	39.3%	51.4%	27.8%	30.4%	39.8%	39.1%	44.1%	49.9%

^1^43 individuals did not provide information about their course status

**Table 2:** The score of PC and search skills of medical (M)/dental (D) students by year of study

**Year of study**	**M1**	**D1**	**M2**	**D2**	**M3**	**D3**	**M4**	**D4**	**M5**	**D5**	**M6**	**All D**	**All M**
N	67	24	60	17	55	14	44	19	32	7	37	81	295
Mean score of PC skills (SD)	26.3 (9.8)	27.2 (8.8)	24.2 (9.5)	25.6 (10.8)	24.6 (9.5)	18.4 (9.3)	25.2 (9.7)	18.3 (9.4)	25.8 (8.6)	21.0 (9.4)	26.1 (10.1)	22.7 (9.9)	25.3 (9.5)
P	n.s.		n.s.		0.033		0.011		n.s.		--	0.000	--
t/df	-3.80/86		-.527/74		2.174/65		2.620/61		1.404/36			2.139/366	
													
Mean score of search skills (SD)	19.0 (6.7)	18.7 (6.3)	17.4 (6.4)	13.7 (7.9)	17.3 (7.2)	16.1 (9.9)	19.3 (6.0)	13.2 (4.8)	19.2 (6.0)	13.1 (5.9)	20.6 (6.8)	15.4 (7.3)	18.7 (6.7)
P	n.s.		n.s.		n.s.		0.000		0.023		--	0.033	
t/df	.191/88		1.99/75		.528/66		3.793/60		2.365/37			3.774/371	

**Table 3:** The proportion (%) of medical (M)/dental (D) students' (N = 295, N = 81) use of MEDLINE and full-text articles by year of study

Year of study	**M1**	**M2**	**M3**	**M4**	**M5**	**M6**	**All M**	**D1**	**D2**	**D3**	**D4**	**D5**	**All D**
searching MEDLINE at least 2–7 times/month for study purposes	41.8%	10.0%	18.2%	6.8%	40.6%	35.1%	24.8%	45.8%	5.9%	7.1%	10.5%	14.3%	19.8%
searching MEDLINE at least 2–7 times/month for research	9.2%	23.3%	37.0%	44.2%	40.6%	62.8%	32.5%	21.7%	11.8%	14.3%	36.8%	57.1%	24.1%
not using MEDLINE at all	4.6%	20.0%	13.0%	16.3%	12.5%	5.7%	15.0%	8.7%	41.2%	21.4%	0	0	14.8%
use of full-text journal articles at least 2–7 times/month	16.7%	18.3%	40.0%	29.6%	48.4%	43.2%	33.1%	8.3%	5.9%	7.1%	10.6%	28.6%	10.0%
not using full-text at all	48.5%	46.7%	36.4%	43.2%	29.0%	21.6%	39.6%	37.5%	76.5%	35.7%	47.4%	42.9%	48.1%

**Table 4:** Bivariate correlation coefficients between explanatory and outcome variables among 403 students

		5	4	3	2	1
1	Score of PC skills ^1^	.204	.184	.171	.606	-
2	Score of search skills ^1^	.459	.379	.379	-	-
3	Searching Medline for study purposes ^2^	.328	.273	-	-	-
4	Searching Medline for research ^2^	.584	-	-	-	-
5	Use of full-text journal articles ^2^	-	-	-	-	-

^1^explanatory variable

^2^outcome variable

All correlation coefficients are significant p < .01

**Table 5:** The association between use of MEDLINE/full-text articles and the score of skills of PC use and information searching among 285 students; three binary logistic regression models A, B and C, adjusted for duration of studies

**A. Use of MEDLINE in studies (yes/no)**	*Relative Risk*	*95% CI*	*p*
Gender (female vs. male)	1.83	0.93–3.68	ns
Search skills^1^	1.00	---	
intermediate	2.67	1.26–5.63	0.01
highest	5.90	2.33–14.9	0.000
PC skills ^1^	1.00	---	
Intermediate	1.61	0.44–3.59	ns
Highest	1.06	0.44–2.51	ns
Medical student (medical vs. dental)	0.87	0.39–1.90	ns
			
**B. Use of MEDLINE in research (yes/no)**	*Relative Risk*	*95% CI*	
Gender (female vs. male)	1.83	0.98–3.43	ns
Search skills^1^	1.00	---	
intermediate	1.34	0.69–2.59	ns
highest	4.32	2.03–9.20	0.000
PC skills ^1^	1.00	---	
Intermediate	2.00	1.01–3.97	0.045
Highest	2.68	1.23–1.97	0.013
Medical student (medical vs. dental)	0.95	0.48–1.84	ns
			
**C. Use of full-text articles (yes/no)**	*Relative Risk*	*95% CI*	
Gender (female vs. male)	1.75	0.93–3.27	ns
Search skills^1^	1.00	----	
intermediate	6.18	3.03–12.59	0.000
highest	13.94	6.07–32.0	0.000
PC skills ^1^	1.00	----	
Intermediate	0.72	0.35–1.50	ns
Highest	0.67	0.29–1.51	ns
Medical student (medical vs. dental)	1.86	0.94–3.68	ns

^1 ^Scales were divided in tertiles separately for medical and dental students (lowest/intermediate/highest level)

**Table 6:** The proportion (%) of medical (M)/dental (D) students (N = 295, N = 81) using other medical databases and WWW by year of study

Year of study	**M1**	**M2**	**M3**	**M4**	**M5**	**M6**	**All M**	**D1**	**D2**	**D3**	**D4**	**D5**	**All D**
searching medical portal "Terveysportti" (2+ times/week)	7.5%	8.3%	21.8%	47.7%	50.0%	78.4%	29.8%	4.2%	0.0%	7.1%	15.8%	14.3%	7.4%
use of other medical databases (e.g., Cochrane library or others; yes/no).	37.3%	35.0%	43.6%	45.5%	43.8%	70.3%	44.1%	37.5%	17.6%	35.7%	42.1%	28.6%	33.3%
searching Web for medical information (2+ times/week)	10.6%	10.0%	10.9%	11.4%	12.9%	5.4%	10.2%	20.8%	0.0%	7.1%	5.3%	0.0%	8.6%
using electronic medical handbooks in the medical library (2+ times/month)	14.9%	6.7%	9.1%	6.8%	12.5%	21.6%	11.5%	8.3%	5.8%	21.4%	10.5%	0.0%	9.9%

**Table 7:** Table 7

**1. Use of electronic journals, online databases and search engines**		
**How often do you search the following databases/use the following sources of information?**		
*1. Never*		
*2. Seldom (1–7 times per term)*		
*3. Quite often (2–7 times per month)*		
*4. very often (2+ times per week)*		
MEDLINE (PubMed or OVID) for study purposes	0 1 2 3	
MEDLINE (PubMed or OVID) for research	0 1 2 3	
The EBM and other clinical databases of the 'Terveysportti' portal	0 1 2 3	
Other medical databases (e.g. Cochrane library)	0 1 2 3	
Electronic medical handbooks in the medical library	0 1 2 3	
Full-text journal articles online	0 1 2 3	
Search the Web for professional medical information	0 1 2 3	
**2. Computer literacy and seach skills**		
**Rate your proficiency in general computing skills and information-searching skills**		
*0. have not used the application*		
*0. don't know how to use it*		
*1. some elementary skills*		
*2. I can use, but would learn more*		
*3. my skills are OK*		
*General computing skills*		
Basic use of Windows	0 0 1 2 3	
Advanced Web browsing	0 0 1 2 3	
Advanced use of printers	0 0 1 2 3	
Advanced word processing	0 0 1 2 3	
Presentation graphics (PowerPoint)	0 0 1 2 3	
Use of spreadsheet (Excel)	0 0 1 2 3	
Statistical software	0 0 1 2 3	
*Information searching skills*		
Searching MEDLINE (OWG/Ovid)	0 0 1 2 3	
Searching MEDLINE (PubMed)	0 0 1 2 3	
Use of EBM databases of 'Terveysportti' portal	0 0 1 2 3	
Use of Cochrane library	0 0 1 2 3	
Use of full-text articles	0 0 1 2 3	
Use of electronic textbooks	0 0 1 2 3	
Use of students' digital learning material deposit	0 0 1 2 3	
Advanced use of WWW search engines to obtain	0 0 1 2 3	
professional medical information		
**3. Improvement of computing skills**		
**Which training programs you would like to participate in for improving your skills?**		
Use of electronic textbooks	yes	no
Use of electronic full-text articles	yes	no
Library's news alert for novel scientific publications	yes	no
Web of Science -citation database	yes	no
RefWorks for managing references	yes	no
SPSS software	yes	no
Advanced use of PDF documents (reading, filing, printing)	yes	no
Advanced home use of licensed scientific databases	yes	no

^1 ^Scales were divided in tertiles separately for medical and dental students (lowest/intermediate/highest level)
